# Cannabinoids reduce ErbB2-driven breast cancer progression through Akt inhibition

**DOI:** 10.1186/1476-4598-9-196

**Published:** 2010-07-22

**Authors:** María M Caffarel, Clara Andradas, Emilia Mira, Eduardo Pérez-Gómez, Camilla Cerutti, Gema Moreno-Bueno, Juana M Flores, Isabel García-Real, José Palacios, Santos Mañes, Manuel Guzmán, Cristina Sánchez

**Affiliations:** 1 Dept. Biochemistry and Molecular Biology I, School of Biology, Complutense University, Madrid, Spain; 2 Dept. Immunology and Oncology, Centro Nacional de Biotecnología, Madrid, Spain; 3 Dept. Biochemistry, School of Medicine, Universidad Autónoma-Instituto de Investigaciones Biomédicas "Alberto Sols", IdiPaz, Madrid, Spain; 4 Dept. Animal Surgery and Medicine, School of Veterinary, Complutense University, Madrid, Spain; 5 Servicio de Anatomía Patológica, Hospital Virgen del Rocío, Seville, Spain; 6 Current Address: MMC, Dept. Pathology, University of Cambridge, United Kingdom; CC, Dept. Life Sciences, The Open University, Milton Keynes, UK

## Abstract

**Background:**

ErbB2-positive breast cancer is characterized by highly aggressive phenotypes and reduced responsiveness to standard therapies. Although specific ErbB2-targeted therapies have been designed, only a small percentage of patients respond to these treatments and most of them eventually relapse. The existence of this population of particularly aggressive and non-responding or relapsing patients urges the search for novel therapies. The purpose of this study was to determine whether cannabinoids might constitute a new therapeutic tool for the treatment of ErbB2-positive breast tumors. We analyzed their antitumor potential in a well established and clinically relevant model of ErbB2-driven metastatic breast cancer: the MMTV-neu mouse. We also analyzed the expression of cannabinoid targets in a series of 87 human breast tumors.

**Results:**

Our results show that both Δ^9^-tetrahydrocannabinol, the most abundant and potent cannabinoid in marijuana, and JWH-133, a non-psychotropic CB_2 _receptor-selective agonist, reduce tumor growth, tumor number, and the amount/severity of lung metastases in MMTV-neu mice. Histological analyses of the tumors revealed that cannabinoids inhibit cancer cell proliferation, induce cancer cell apoptosis, and impair tumor angiogenesis. Cannabinoid antitumoral action relies, at least partially, on the inhibition of the pro-tumorigenic Akt pathway. We also found that 91% of ErbB2-positive tumors express the non-psychotropic cannabinoid receptor CB_2_.

**Conclusions:**

Taken together, these results provide a strong preclinical evidence for the use of cannabinoid-based therapies for the management of ErbB2-positive breast cancer.

## Background

Breast cancer represents approximately 30% of newly diagnosed cancers each year. Almost one third of them overexpresses the ErbB2 tyrosine kinase receptor (Her2 in humans, Neu in rats), a member of the EGF receptor family [1]. Phosphorylation of their intracellular domains upon engagement by their ligands induce receptor homo- or heterodimerization, leading to the activation of key signaling pathways that promote cell proliferation and survival, including the phosphatidylinositol 3-kinase (PI3K)/Akt pathway and the ERK/MAPK cascade. Although no specific ligand for ErbB2 has been identified yet, this receptor is the preferred heterodimerization partner of the family [2]. ErbB2-overexpressing breast tumors are characterized by very aggressive clinical courses and decreased survival rates, mostly due to the poorly differentiated, highly proliferative and highly invasive nature of their constituent cells [2]. All these characteristics make ErbB2-overexpressing tumors less responsive to conventional therapies. One of the most recent advances in the treatment of these tumors is the use of a humanized neutralizing monoclonal antibody against ErbB2 (Trastuzumab) [3]. Although this strategy has been very successful, around 75% of patients with ErbB2-overexpressing tumors do not respond to Trastuzumab, and nearly 15% of the responders eventually develop metastases [4]. The existence of this considerable population of non-responding and relapsing patients urges the search for novel treatments.

The therapeutic potential of cannabinoids, the active compounds of marijuana and their derivatives, has been known for centuries. There is increasing evidence supporting that they might be beneficial in various pathological contexts such as pain, inflammation, eating disorders, and brain damage, amongst others [5,6]. Cannabinoids exert most of their actions by binding to and activating specific G protein-coupled receptors. To date, two cannabinoid receptors, namely CB_1 _and CB_2_, have been cloned and characterized from mammalian tissues, the main difference between them being their tissue expression pattern. Thus, while CB_1 _receptors are ubiquitously located, with their highest presence found in the central nervous system, CB_2 _receptor expression is mostly restricted to particular elements of the immune system [5,6]. During the last decade, evidence has accumulated suggesting that cannabinoids might be useful for the treatment of cancer. These compounds exert anti-proliferative, pro-apoptotic, anti-angiogenic, and anti-invasive effects in different cell-culture and animal models of cancer [7,8]. Here, we used a genetically engineered animal model of ErbB2-driven metastatic breast cancer (the MMTV-neu mouse) to analyze the antitumoral potential of cannabinoids in this particularly aggressive pathology. These animals express the rat ErbB2 oncogene (neu) under the control of the hormone-sensitive mouse mammary tumor virus-long terminal repeat (MMTV-LTR) promoter [9]. Selective overexpression of neu in the mammary epithelium results in the spontaneous development of focal mammary tumors after a long latency (5-12 months) [9]. Results presented herein (i) show that ErbB2-positive invasive human breast tumors express CB_2 _receptors, (ii) demonstrate that Δ^9^-tetrahydrocannabinol (THC) and the non-psychotropic CB_2 _cannabinoid receptor agonist JWH-133 significantly reduce tumor progression in a clinically relevant model of ErbB2-positive metastatic breast cancer, and (iii) shed light on the mechanism of cannabinoid antitumoral action *in vivo*.

## Results

### Human ErbB2-positive breast tumors express CB_2 _cannabinoid receptors

**Figure 1:**
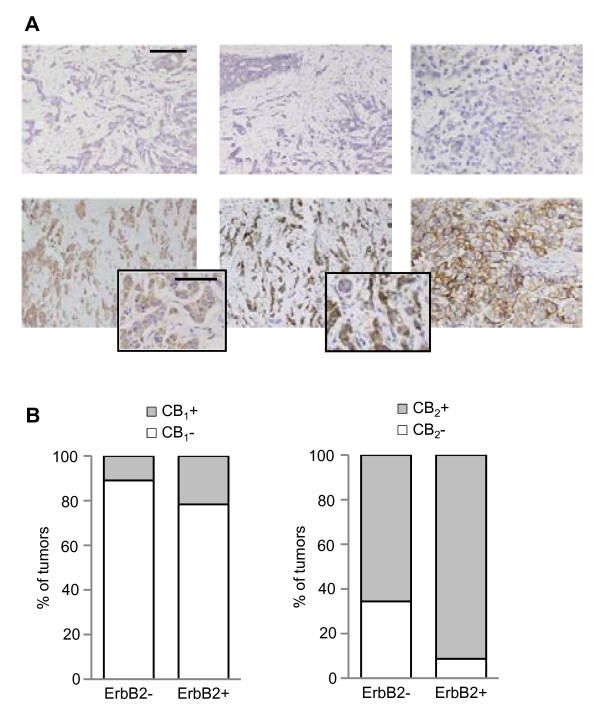
**ErbB2-positive human breast tumors express cannabinoid receptors**. (A) Representative images of human breast tumors negative (upper row) or positive (lower row) for CB_1_, CB_2 _and ErbB2 receptors (brown). Scale bars: 200 μm; insets: 100 μm. (B) Percentage of tumors scored as positive or negative for cannabinoid receptor expression amongst the ErbB2-negative (n = 64) and ErbB2-positive (n = 23) populations.

We first analyzed whether ErbB2-positive human breast tumors express cannabinoid targets (i.e. cannabinoid receptors). We performed an immunohistochemical analysis of CB_1 _and CB_2 _receptors in 87 grade 3 invasive breast ductal carcinomas and 6 non-tumoral mammary samples by tissue microarrays. CB_1 _immunoreactivity was detected in only 14% of the tumors (12/87), and no correlation was found between this receptor expression and ErbB2 expression (p = 0.198, Fig. 1). Conversely, CB_2 _receptor staining was evident in 72% of the carcinomas (63/87) and it was significantly associated with ErbB2 expression, since it was observed in 91% of the ErbB2-positive tumors (21/23, p = 0.018, Fig. 1). Moreover, we detected no significant CB_1 _or CB_2 _receptor immunoreactivity in non-transformed mammary tissue (data not shown).

### Cannabinoids exert an antitumoral effect in the MMTV-neu model of breast cancer

**Figure 2:**
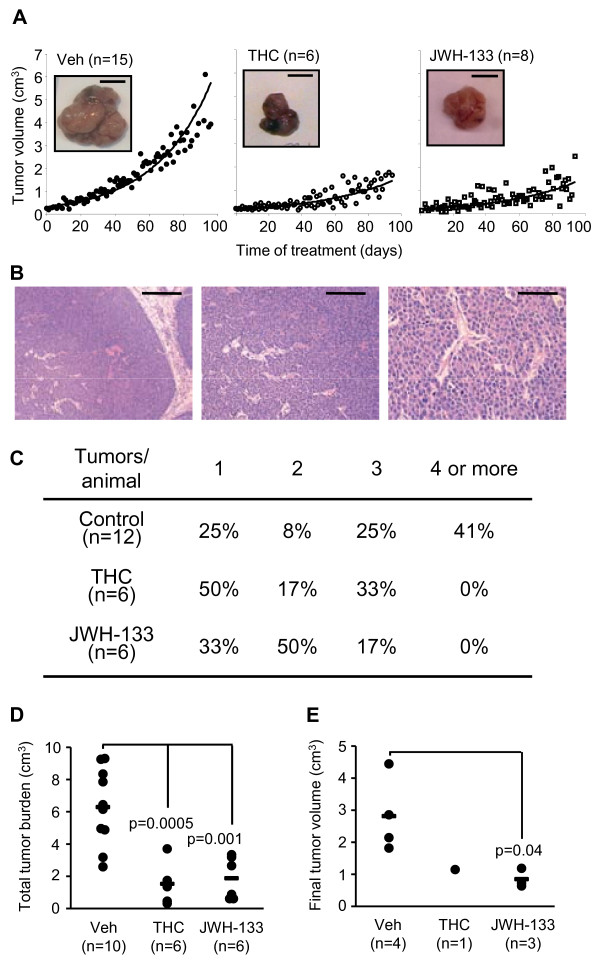
**Cannabinoids inhibit breast tumor growth *in vivo *and the number of tumors generated per animal**. (A) Volume time-course (scale bar: 1 cm) of the first tumor appeared in each animal. (B) Representative images (H&E staining) of the histopathology of the MMTV-neu-derived mammary tumors. Scale bars (from left to right): 200 μm, 100 μm and 50 μm. (C) Percentage of animals with 1, 2, 3, 4 or more tumors at the end of the treatment (90 days) in each experimental group. (D) Total tumor burden (total tumor volume per animal) determined 90 days after cannabinoid or vehicle treatment. (E) Volume of the tumors appeared in second, third or subsequent positions, 40 days after their appearance. The small size of the cannabinoid-treated groups is due to the very few second or third tumors appeared early enough to last 40 days in the animals before the end of the treatment (90 days after the appearance of the first tumor).

We then analyzed the effect of cannabinoids on tumor progression in a well established and clinically relevant animal model of ErbB2-driven metastatic breast cancer, the MMTV-neu mouse. We first observed that our MMTV-neu colony develops breast tumors after a long latency period similar to that previously reported [9]. In particular, 50% of the females had tumors by week 36 (Additional file 1: Fig. S1A). Overexpression of the rat ErbB2 transgene (neu) in the tumors was verified by real-time quantitative PCR (Additional file 1: Fig. S1B). Treatment with cannabinoids, either THC, the main marijuana-derived cannabinoid in terms of abundance and potency, or JWH-133, a synthetic CB_2 _receptor-selective agonist, strongly slowed down tumor growth (Fig. 2A), leading to smaller lesions at the end of the treatment (Additional file 1: Fig. S2). These compounds, however, did not change the histomorphologic features of the tumors. Thus, the three different experimental groups generated focal, ductal, solid, well vascularized mammary tumors surrounded by a non-invasive hyperplasic mammary epithelium (Fig. 2B). We also observed that MMTV-neu-derived tumors express CB_1 _and CB_2 _cannabinoid receptor mRNA (determined by real-time quantitative PCR, data not shown) and protein (Additional file 1: Fig. S3).

Of interest, cannabinoids not only impaired tumor growth, but also blocked tumor generation *per se*. Thus, while 41% of vehicle-treated animals developed 4 or more tumors (up to 6), cannabinoid-treated animals never developed more than 3 tumors (Fig. 2C, p < 0.05). Consequently, total tumor burden was strikingly decreased by cannabinoids (Fig. 2D). There was also a delay in the appearance of the subsequent tumors in these animals. Thus, the average latency for the generation of a second tumor in vehicle-treated, THC-treated and JWH-133-treated animals was 33, 46 and 54 days, respectively. As mentioned in the Methods section, only the first tumor in each animal was treated peritumorally with cannabinoids. However, we detected a remarkable growth-inhibitory effect of cannabinoids in those tumors appeared in second place (Fig. 2E).

### Cannabinoids impact tumor cell proliferation, tumor cell survival and tumor angiogenesis

**Figure 3:**
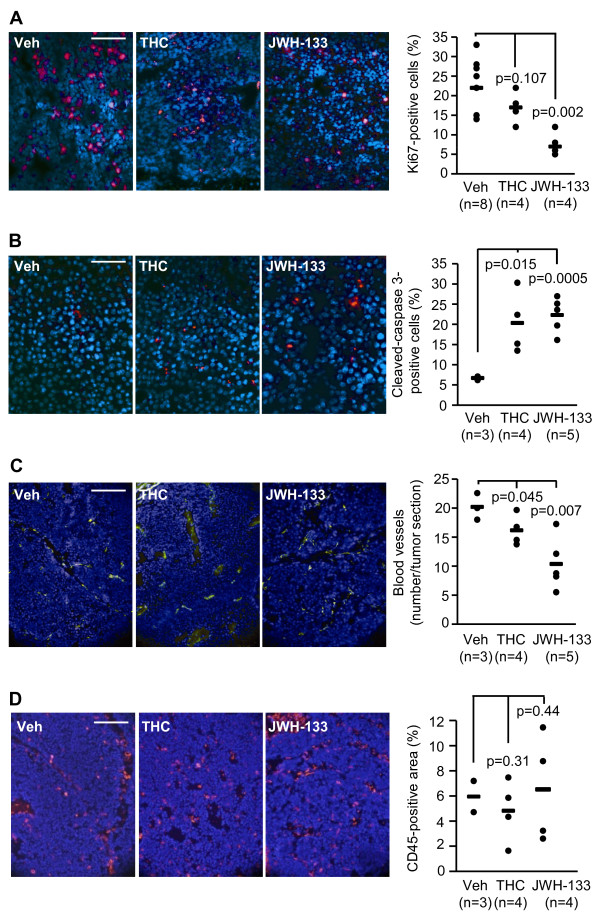
**Cannabinoids inhibit cancer cell proliferation, induce cancer cell apoptosis, and impair tumor angiogenesis *in vivo***. (A) Ki67-positive cells (red), (B) active caspase-3-positive cells (red), (C) CD31-positive cells (green) and (D) CD45-positive area in the tumors. Scale bars: A, 60 μm; B, 40 μm; C and D, 100 μm. Cell nuclei are in blue. Quantifications of Ki67-positive cells (A), active caspase-3-positive cells (B), the number of blood vessels (C) and CD45-positive area (D) in the tumors are shown in the corresponding graphs.

We next analyzed the proliferative potential of cancer cells and found that it was reduced by both THC and JWH-133, as indicated by a decreased number of Ki67-positive cells in cannabinoid-treated tumors (Fig. 3A). Cannabinoid administration also increased the number of cleaved (active) caspase 3-positive cells within the tumors, indicating that these compounds induce cancer cell death by apoptosis (Fig. 3B). Tumor vascularization was also impaired by cannabinoids, as both THC and JWH-133 decreased the number of blood vessels in the tumors, as determined by CD31 staining (Fig. 3C). To evaluate the possible contribution of the immune response to cannabinoid antitumoral action, we analyzed by immunofluorescence the degree of immune infiltration in the tumors. The percentage of CD45-positive cells (differentiated hematopoietic cells except erythrocytes and platelets) within the tumors was very low in all the samples tested and no significant differences between experimental groups were detected (Fig. 3D). These data suggest that cannabinoid treatment does not affect the infiltration of immune cells into the tumor parenchyma.

### Cannabinoids decrease breast cancer metastases in the lungs

**Figure 4:**
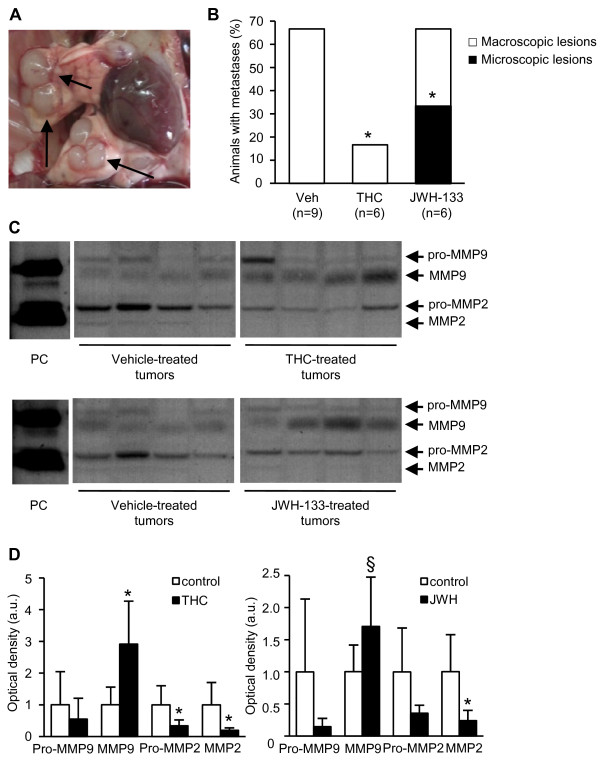
**Cannabinoids inhibit breast cancer metastasis to the lungs *in vivo***. (A) Metastatic lung nodules (pointed by arrows). (B) Percentage of animals with lung metastases. Macroscopic metastases were visible to the naked eye and microscopic lesions were detectable only by H&E staining. These latter lesions were found only in JWH-133-treated animals. (C) Gelatin zymographies of vehicle- and cannabinoid-treated tumors. Four representative tumors are shown per experimental group. PC: positive control (conditioned medium of H71080 cells stimulated with the phorbol ester PMA). Arrows point to the latent (pro-MMP) and active MMP bands according to the positive control and the expected MMP molecular weights. Non-contiguous parts of the same gel are shown. Graphs show the densitometric analysis of MMP2 and MMP9 activities. Data are expressed in arbitrary units. *, p < 0.05 *vs *vehicle-treated tumors; §, p = 0.068 *vs *vehicle-treated tumors.

It has been previously reported that a high percentage of tumor-bearing MMTV-neu animals develop metastases in the lungs [9]. Specifically, we detected lung metastases (Fig. 4A) in 67% of our vehicle-treated MMTV-neu animals (Fig. 4B). The cell morphology, tumor architecture, and overexpression of the neu transgene mRNA in these lung structures confirmed the metastatic nature of the lesions (Figs. S1C and D). THC reduced the percentage of animals with lung metastases (Fig. 4B). Although JWH-133 did not decrease this proportion, it significantly reduced the magnitude of the lesions. Thus, half of the metastases in this experimental group were detectable only by microscopic analysis (Fig. 4B). As it was observed for the primary breast tumors, cannabinoid treatment did not alter the histopathology of the metastases, and the three experimental groups presented similar solid adenocarcinomas (Additional file 1: Fig. S1C). No sign of metastasis was detected in any of the other organs analyzed (brain, spleen, liver, kidneys -by histological analysis- and bones -by X-rays) in any of the experimental groups (data not shown).

Degradation of the extracellular matrix is a crucial step in the metastatic process, especially during tumor cell intravasation and extravasation [10]. Matrix metalloproteinases (MMPs) have long been associated with this process owing to their ability to degrade the components of the extracellular matrix. To analyze whether cannabinoid administration affects MMP activity we conducted gelatin zymographies. MMP2 activity was decreased in THC- and JWH-133-treated tumors, while MMP9 activity was enhanced by cannabinoid treatment (Fig. 4C). This THC- and JWH-133-induced reduction in MMP2 activity was accompanied by a decrease in MMP2 mRNA levels (Additional file 1: Fig. S4A). Conversely, cannabinoids did not change the amount of MMP9 transcripts (Additional file 1: Fig. S4A) and enhanced its protein levels (Additional file 1: Fig. S4B), indicating that they regulate MMP9 post-transcriptionally.

### Akt downregulation is involved in cannabinoid antitumoral action

**Figure 5:**
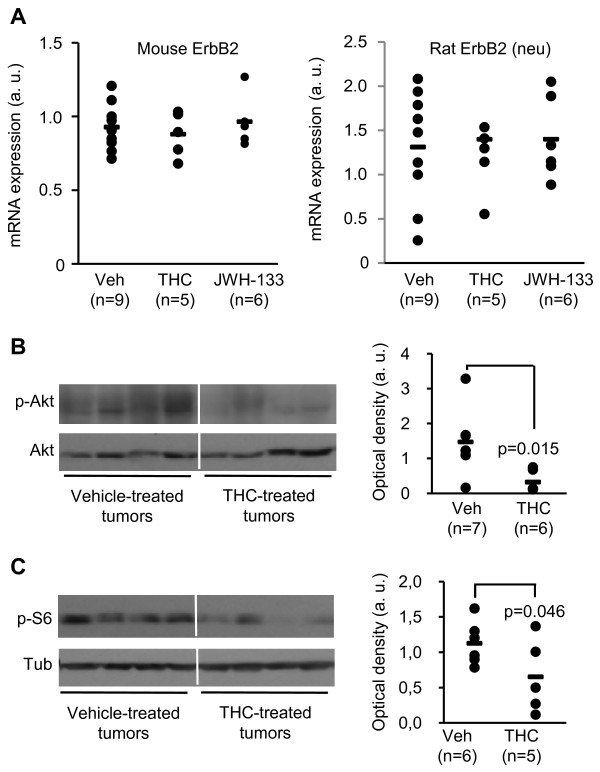
**THC inhibits Akt *in vivo***. (A) Mouse ErbB2 and (B) rat ErbB2 (neu transgene) mRNA expression in vehicle- and cannabinoid-treated tumors as determined by real-time quatitative PCR. (B and C) Western blot and densitometric analysis of phospho-Akt (B) and phospho-S6 ribosomal protein (C) levels in MMTV-neu-derived tumors treated with vehicle or THC. Eight representative tumors are shown. Total Akt (B) and α-tubulin (C) were used for normalization. Non-contiguous parts of the same gel are shown. Optical densities are expressed in arbitrary units.

We next aimed at characterizing the mechanism underlying cannabinoid antitumoral effect. It is well established that several types of human cancers are associated with deregulation of signaling via ErbB members [1]. In particular, ErbB2 overexpression correlates, for instance, with tumor size, increased metastatic potential, and higher histological grade, implying that ErbB2 confers a strong proliferative and survival advantage to tumor cells [11]. To assess whether cannabinoids modulate the expression of endogenous ErbB2 and of the rat ErbB2 ortologue neu, which is ectopically expressed in our animal model, we conducted real-time quantitative PCR determinations upon THC and JWH-133 treatment. However, no significant changes were detected (Fig. 5A).

**Figure 6:**
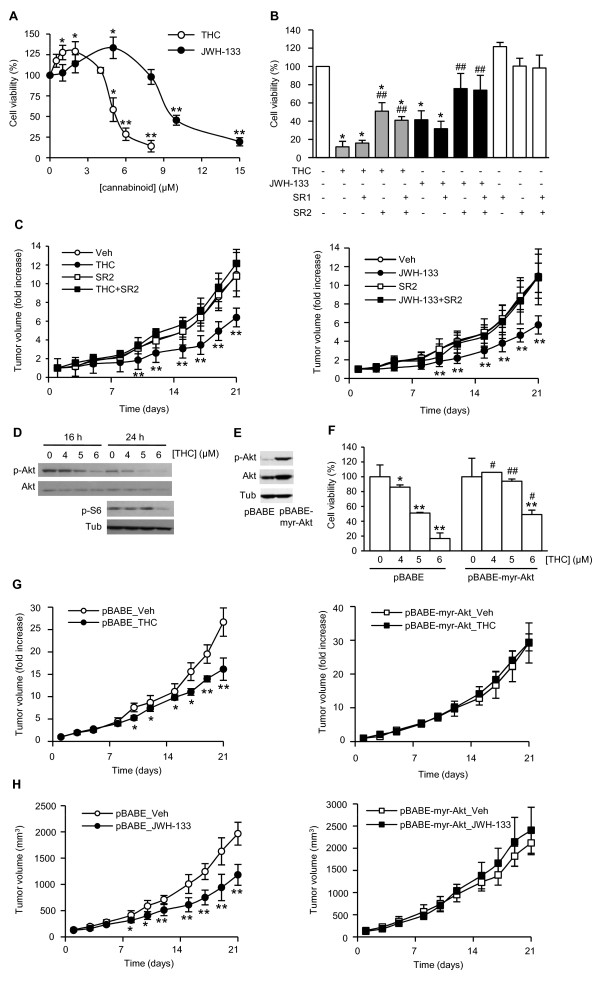
**Akt downregulation is involved in cannabinoid antitumoral action**. (A and B) Viability of N202.1A cells in response to (A) increasing concentrations of THC or JWH-133 or (B) 6 μM THC or 10 μM JWH-133 with or without 2 μM SR141716 (SR1) and/or SR144528 (SR2) for 48 h. Data are expressed as % of vehicle-treated cells, set at 100%. (C) Growth of N202.1A-derived xenografts treated with THC (left panel) or JWH-133 (right panel) with or without SR2. (D) Phospho-Akt and phospho-S6 ribosomal protein (p-S6) levels in N202.1A cells challenged with THC, as determined by Western blot. Total Akt and α-tubulin levels were used for normalization. (E) Phospho-Akt and total Akt levels in N202.1A cells retrovirally transduced with myristoylated Akt (pBABE-Myr-Akt) or the corresponding empty vector (pBABE). (F) Cell viability of pBABE- or myr-Akt-transduced N202.1A cells in response to THC exposure for 72 h. (G and H) Time course of the volume of pBABE-N202.1A-derived (left panels) and myr-Akt-N202.1A-derived (right panels) tumors treated with THC (G), JWH-133 (H) or the corresponding vehicle. *, p < 0.05; **, p < 0.01 *vs *vehicle-treated cells or tumors; #, p < 0.05; ##, p < 0.01 *vs *cannabinoid alone-treated cells (B) or *vs *THC-treated pBABE-transduced cells (F).

A central intracellular signaling pathway activated by ErbB2 is the PI3K/Akt pathway, whose importance in breast cancer is corroborated by clinical studies showing Akt activation in most ErbB2-overexpressing tumors [11]. We observed a decrease in Akt activation in THC-treated MMTV-neu tumors (Fig. 5B) as well as diminished levels of phosphorylated S6 ribosomal protein [a read-out for activation of the Akt/mammalian target of rapamycin (mTOR) pathway [12]] (Fig. 5C). To determine the importance of Akt inhibition in cannabinoid antitumoral action we conducted different experiments with the cell line N202.1A, that was isolated from a MMTV-neu breast tumor [13]. THC and JWH-133 decreased N202.1A cell proliferation (Fig. 6A) in a CB_2 _receptor-dependent manner, as indicated by the prevention of cannabinoid action exerted by the CB_2 _receptor-selective antagonist SR144528 but not by the CB_1 _receptor-selective antagonist SR141716 (Fig. 6B). Likewise, the growth rate of N202.1A-derived xenografts was significantly diminished by THC and JWH-133, and this effect was prevented by SR144528 (Fig. 6C). THC also decreased cell proliferation of two different ErbB2-overexpressing breast cancer cell lines of human origin (Additional file 1: Fig. S5), suggesting that human ErbB2-positive breast tumor cells may be sensitive to cannabinoid antitumoral action as well. N202.1A cells showed a dose-dependent reduction in Akt activation (Fig. 6D). Of interest, overexpression of a myristoylated (i.e. constitutively activated) form of Akt (Fig. 6E) prevented THC antiproliferative effect (Fig. 6F). To further support the importance of Akt in cannabinoid antitumoral action, subcutaneous xenografts were generated in nude mice with N202.1A cells stably expressing myristoylated Akt or the corresponding empty vector (pBABE). As shown in Fig. 6G (left panel), THC significantly reduced the growth of pBABE-transfected N202.1A-derived tumors. In contrast, overexpression of activated Akt prevented THC effect on tumor progression (Fig. 6G, right panel). The same effect was observed with JWH-133 (Fig. 6H).

## Discussion

Aberrant ErbB2 expression and/or function yield highly aggressive tumors, with increased resistance to conventional chemotherapies and poor outcomes. Although the use of the anti-ErbB2 monoclonal antibody Trastuzumab markedly improves the survival of these patients, only 25% of them respond to this treatment and most of the responders eventually relapse [14]. Moreover, the use of this antibody has been associated with important cardiotoxic side effects (severe congestive heart failure and decrease in left ventricular ejection fraction) [14]. Consequently, extensive efforts should be made to find novel agents for the treatment of ErbB2-positive breast tumors. Our results demonstrate that, in spontaneously aroused ErbB2-overexpressing breast tumors, cannabinoids inhibit tumor generation, growth, vascularization, and metastasis. Although a cannabinoid-based monotherapy might be potentially effective for ErbB2-positive breast tumors, it would be interesting to analyze the effect of these compounds in combination with other anticancer treatments. Thus, it is worth noting that Trastuzumab, the most relevant targeted therapy for ErbB2-positive tumors so far, has a modest median overall response when used as a first-line agent, an efficacy that is clearly enhanced when used in combination with other chemotherapeutic agents [14]. Additionally, Akt overactivation has been detected in a significant percentage of primary human breast cancers, in which it is associated to enhanced resistance to Trastuzumab [14,15]. Our results show that downregulation of Akt is involved in cannabinoid antitumoral action. This kinase is the central node of the PI3K/Akt/mTOR signaling pathway, that activates crucial processes such as cell survival, cell growth, cell proliferation, angiogenesis, and cell migration and invasion [12]. This pathway is therefore an attractive target for anticancer agents and, as a matter of fact, clinical trials have been/are being conducted with mTOR, PI3K and Akt inhibitors [3].

The antitumoral potential of cannabinoids has been documented both *in vitro *and in animal models of cancer [7,8]. These compounds inhibit breast cancer cell proliferation *in vitro *through processes that include cell cycle arrest [16-21], hormone and growth-factor receptor modulation [18,22,23], and apoptosis induction [17,20,21]. The *in vivo *approaches followed so far have been mostly based on xenograft models [20,21], which are helpful but limited tools. These models rely on the propagation of cancer cell lines in immunodeficient mice at ectopic or orthotopic sites and lack crucial features of patients' tumors such as the actual tumor architecture and the interactions with the tumor microenvironment (including non-cancerous surrounding tissue, vasculature and immune cells) and diminished genetic heterogeneity [24]. In contrast to xenografted animals, in the mutant mice used in this study tumors appear spontaneously and after long latency periods, recruit and generate blood vessels, and penetrate the vasculature giving rise to distant metastases [9]. These features parallel the human pathology much more closely and make the MMTV-neu mice a clinically relevant model of ErbB2-driven breast cancer.

Remarkably, this is, to the best of our knowledge, the first report supporting that cannabinoids hamper not only tumor growth but also tumor generation. Recently, Qamri and coworkers and DuBois and coworkers, by using two different genetic models of cancer, demonstrated that JWH-133 delays the appearance of breast tumors [21] and that the loss of CB_1 _receptors accelerates intestinal adenoma growth [25], respectively, and Izzo *et al*. observed that high endocannabinoid levels and the cannabinoid agonist HU-210 reduce the development of precancerous lesions in the mouse colon [26]. These and our data suggest that the endocannabinoid system has a physiological protective role against tumorigenesis, in line with the general idea that this system contributes to maintain homeostasis in health and disease [6].

Data presented herein show that cannabinoids modulate MMP activity. In particular, we report an inhibition of MMP2 and an activation of MMP9 by cannabinoids. Although MMPs have been traditionally associated to metastasis due to their ability to degrade the extracellular matrix, it has been recently shown that several members of this family provide a protective effect in different stages of cancer progression [27]. One of these antitumoral MMPs is MMP9, which is activated by cannabinoids in our system. Although this MMP may promote the angiogenic switch in some experimental tumors [e.g. [28]], clinical studies have established a correlation between MMP9 overexpression and good prognosis in breast cancer [29]. This protective effect might derive from its capacity to generate angiogenesis inhibitors such as angiostatin and tumstatin [27]. The inhibition of MMP2 by cannabinoids shown here, in line with that previously reported by Bifulco and coworkers in thyroid cancer cells [30] and our group in gliomas [31], may be of special relevance considering that high tumor levels of this metalloproteinase have been correlated with poor prognosis in breast cancer [32]. In addition, enhanced levels of MMP2 in breast tumors are associated with ErbB2 gene amplification and/or overexpression [33]. Moreover, Massagué and coworkers have recently identified MMP2 as one of the genes of the signature that mediates breast cancer metastasis to the lungs [34], the targeted metastatic organ in our animal model.

Potential antitumoral therapies based on the use of cannabinoids might be limited by their well known psychotropic actions such as dizziness, dry mouth, tiredness, muscle weakness, euphoria, myalgia and palpitations [6,35]. Although the benefit/risk ratio is potentially high for cannabinoid-based therapies, different strategies should be taken to avoid or at least minimize their side effects. Since most -if not all- of the psychoactive effects of cannabinoids are produced by the activation of central CB_1 _receptors [5,6], one reasonable approach would be targeting CB_2 _receptors selectively. Here, we have demonstrated that the CB_2_-selective agonist JWH-133 is as effective as THC (a CB_1_/CB_2_-mixed agonist) in reducing tumor generation and progression. Moreover, our results also (i) show that an elevated percentage of high grade ErbB2-positive human breast tumors express CB_2 _receptors, and (ii) that a very low fraction of them express CB_1 _receptors. Taken together, these data suggest that activation of CB_2 _in this particular population of patients would be an efficient strategy to treat breast tumors without triggering psychoactive effects. A correlation between tumor aggressiveness and CB_2 _receptor expression in breast cancer has been previously reported: tumors lacking estrogen or progesterone receptors, which are associated to low response rates to adjuvant therapies, express higher CB_2 _levels than steroid receptor-positive lesions [17]. Moreover, ErbB2-positive tumors also have increased CB_2 _receptor mRNA levels compared to their less aggressive and more responsive ErbB2-negative counterparts [17]. Of interest, this receptor is scarcely expressed in non-transformed mammary tissue [data presented here and [17,21]].

## Conclusions

In summary, our results, which were obtained in a clinically relevant animal model of ErbB2-positive breast cancer, suggest that these highly aggressive and low responsive tumors could be efficiently treated with non-psychoactive CB_2_-selective agonists without affecting the surrounding healthy tissue.

## Methods

### Tissue microarray analysis

Eighty seven grade 3 invasive breast ductal carcinomas and 6 non-tumoral mammary tissues were fixed in 10% paraformaldehide (PFA) and embedded in paraffin. Two representative tissue cores (1 mm of diameter) of each one were included in a tissue microarray. The main clinical pathology and molecular features of this series had been previously reported [36]. Tissue sections were subjected to a heat-induced antigen retrieval step prior to exposure to the primary antibody. Anti-CB_1 _receptor antibody was generously donated by Dr. Ken Mackie, Indiana University, Indiana, and anti-CB_2 _receptor antibody was from Affinity Bioreagents/Thermo Fisher Scientific, Rockford, Illinois. ErbB2 expression was evaluated using a HercepTest (Dako, Carpenteria, CA) according to manufacturer's instructions. Immunodetection was performed using the LSAB method (DAKO) with DAB as the chromogen. In negative controls, the primary antibody was omitted or replaced with an irrelevant antibody. Cases were reviewed by two independent pathologists (J.P. and G.M-B) and were scored as positive for cannabinoid receptors when more than 25% of the neoplastic cell showed intense immunostaining for the corresponding antibody. ErbB2-staining was scored according to HercepTest manufacturer's guidelines: scores 0 and 1+ were considered as negative, and 2+ and 3+ as positive for ErbB2 overexpression.

### Animals and treatments

All procedures involving animals were performed with the approval of the Complutense University Animal Experimentation Committee according to the European official regulations. FVB/N-Tg(MMTVneu)202 Mul/J mice (more commonly designated as MMTV-neu mice) were obtained from The Jackson Laboratory (Bar Harbor, Maine). Females were palpated twice weekly for mammary gland nodules and cannabinoid treatment was started when the first tumor in each animal was detected. Δ^9^-Tetrahydrocannabinol (THC, The Health Concept, Richelbach, Germany) and JWH-133 (kindly donated by John W. Huffman, Clemson University, South Carolina) were prepared in DMSO (0.2 mg/μL and 0.02 mg/μL, respectively) and diluted in PBS supplemented with 5% BSA (100 μL/dose for tumors ≤ 1000 mm^3 ^and 200 μl/dose for tumors >1000 mm^3^). Cannabinoid peritumoral treatment (0.5 mg THC or 0.05 mg JWH-133/animal/day, twice a week) was maintained for 90 days, and only the first tumor in each animal was treated. Tumors were routinely measured during this period with external caliper, and volume was calculated as (4π/3) × (width/2)^2 ^× (length/2). At the end of the treatment, animals were sacrificed and tumors and organs were collected. Tumors were divided in four portions for 1) preparation of tissue sections for immunofluorescent staining [frozen in Tissue-Tek (Sakura Finetek Europe, Zoeterwoude, The Netherlands)], 2) preparation of tissue sections for hematoxylin-eosin staining (fixed in buffered 4% PFA), 3) protein extraction (snap frozen) and 4) RNA isolation (snap frozen), and were stored at -80°C until analysis (except PFA-fixed tumor fractions, that were kept at room temperature). Brain, spleen, liver, kidneys and lungs were fixed in PFA. For xenograft experiments, subcutaneous tumors were induced in 6 week-old athymic female mice (Harlan Interfauna Iberica, Barcelona, Spain) by subcutaneous injection of 5 × 10^5 ^N202.1A cells. When tumors reached *ca*. 100 mm^3^, they were treated with THC (0.5 mg/animal/day), JWH-133 (50 μg/animal/day), SR144528 (50 μg/animal/day), a combination of cannabinoid and SR144528 or vehicle for 3 weeks, 3 times a week, measured, and processed as described above. For Akt-related experiments, half of the animals were injected with N202.1A cells stably expressing myristoylated Akt (N202.1A-pBABE-myr-Akt), and the other half with N202.1A cells stably expressing the corresponding empty vector (N202.1A-pBABE). Tumors were treated with THC, JWH-133 or vehicle and processed as described for N202.1A xenografts.

### Metastasis detection

Collected organs were visually analyzed for macroscopic metastases. Microscopic metastases were determined by histological analysis of PFA-fixed paraffin-embedded hematoxylin-eosin stained sections. Radiographs were taken to evaluate the presence of bone metastases using a conventional X-ray equipment (Diagnost 93, Philips Medical Systems, Eindhoven, The Netherlands), and mammography cassette (Kodak MIN-R 2000 screen cassette) and film (Kodak MIN-R S film) (Eastman Kodak Company, Rochester, New York).

### Cell culture and viability

N202.1A cells were kindly given by Dr. Vincenzo Bronte (Istituto Oncologico Veneto, Padova, Italy). This cell line was established from a MMTV-neu-derived tumor [13]. BT474, MDA-MB-231, MCF-7 and SkBr3 human breast cancer cells, Jurkat human leukemic cells, and U373 human glioblastoma cells were from ATCC-LGC (Barcelona, Spain). All cell lines were maintained in DMEM supplemented with 10% fetal bovine serum (FBS). Cells were transferred to a low (0.5%)-FBS medium immediately before cannabinoid challenge. Cell viability was determined by the 3-4,5-dimethylthiazol-2,5-diphenyltetrazolium bromide thiazol blue test (Sigma, St. Louis, Missouri) according to manufacturer's instructions.

### Plasmids, transfections and infections

Stable expression of myr-Akt was achieved by retroviral infection. N202.1A cells were transduced for 4 h with supernatants obtained from Phoenix ecotropic cells previously transfected with a retroviral vector carrying HA-tagged myr-Akt (kindly provided by Dr. Pier P. Pandolfi, Harvard University, Boston, Massachusetts) or the corresponding empty construction (pBABE). Infected cells were selected with puromycin.

### Immunofluorescence analysis

Tissue-tek embedded tumor sections were fixed in PFA and incubated with anti-CB_1 _receptor, anti-CB_2 _receptor, anti-CD31 (Pharmingen/BD Biosciences, San Jose, California), anti-CD45 (Pharmingen/BD Biosciences), anti-Ki67 (Neomarkers/Lab Vision, Fremont, California) or anti-cleaved-caspase 3 (Cell Signaling Technology, Danvers, Massachusetts) antibodies. Secondary anti-rabbit antibodies AlexaFluor 594 and AlexaFluor 488 were from Invitrogen (Carlsbad, California). Cell nuclei were stained with Hoescht 33342 (Invitrogen). Fluorescence images were acquired using Metamorph Premier Offline software (Molecular Devices, Sunnyvale, California). Blood vessel size was calculated with ImageJ software.

### Real-time quantitative PCR (RTQ-PCR) and reverse-transcriptase PCR (RT-PCR)

RNA was isolated with Trizol Reagent (Invitrogen), including a DNase digestion step, with the Real Star Kit (Durviz, Valencia, Spain), and cDNA was obtained with Transcriptor Reverse Transcriptase (Roche Applied Science, Penzberg, Germany). The primers used for RTQ-PCR amplification were: mouse CB_1 _receptor, sense 5'-GGGCAAATTTCCTTGTAGCA-3', antisense 5'-GGCTCAACGTGACTGAGAAA-3'; mouse CB_2 _receptor, sense 5'-ATTCAGGAGATCTGTTAAGACAAGG-3', antisense 5'-GACATCTATGAAGTTGAGGCAGTG-3'; mouse MMP2, sense 5'- GCGCTTTTCTCGAATCCAT-3', antisense 5'-GGGTATCCATCTCCATGCTC-3'; mouse MMP9, sense 5'-ACGACATAGACGGCATCCA-3', antisense 5'- GCTGTGGTTCAGTTGTGGTG-3'; rat ErbB2 (neu), sense 5'-GCTCAGAGACCTGCTTTGGA-3', antisense 5'-AGGAGGACGAGTCCTTGTAGTG-3'; mouse ErbB2, sense 5'-AACAGCTCGGAGACCTGCTA-3', antisense 5'-GTAGTGGGCACAAGCCTCA-3'. Probes were from the Universal Probe Library (Roche Applied Science). Multispecies 18S RNA was used as reference (sense 5'- GCTCTAGAATTACCACAGTTATCCAA-3', antisense 5'- AAATCAGTTATGGTTCCTTTGGTC-3'). The primers use for RT-PCR were: mouse MMP2, sense 5'- TCTGCGATGAGCTTAGGGAAAC-3', antisense 5'-GACATACATCTTTGCAGGAGACAAG-3'; mouse MMP9, sense 5'-GGACGACGTGGGCTACGT-3', antisense 5'- CACGGTTGAAGCAAAGAAGGA-3'. GAPDH was used as reference (sense 5'-GGGAAGCTCACTGGCATGGCCTTCC-3', antisense 5'-CATGTGGGCCATGAGGTCCACCAC-3').

### Western blot analysis

Cell lysates from tumors and cell lines were subjected to SDS-PAGE, and proteins transferred onto polyvinylidene fluoride membranes. Blots were incubated with the following antibodiess: anti-CB_1 _receptor, anti-CB_2 _receptor (Affinity Bioreagents), anti-MMP9 (Chemicon International INC, Temecula, California), anti-ErbB2 (Santa Cruz Biotechnology, Santa Cruz, California), anti-phospho-Akt (Ser473), anti-Akt, anti-phospho-S6 ribosomal protein (Cell Signaling) and anti-α-tubulin (Sigma). Luminograms were obtained with the Amersham Enhanced Chemiluminescence Detection Kit (GE Healthcare, Uppsala, Sweden) and densitometric analysis was performed with Quantity One software (Bio-Rad).

### MMP activity assay

MMP2 (Gelatinase A) and MMP9 (Gelatinase B) activities were determined by gelatin zymography. Briefly, SDS-PAGE were run in the presence of 0.1% gelatin, washed with a 2.5% Triton X-100 containing buffer, and incubated overnight at 37°C in 50 mM Tris pH 7.5, 150 mM NaCl, 10 mM CaCl_2_, 0.1% Triton X100. Gels were then stained with Coomasie Blue and digested bands quantified by densitometric analysis as described above.

### Statistical analysis

ANOVA with a post hoc analysis by the Student-Newman-Keuls' test was routinely used. For the analysis of metastases and the number of tumors per animal, a Pearson Х^2 ^test was used. To determine the correlation between immunohistochemical (CB_1 _and CB_2 _expression) and clinical pathology (ErbB2) data, the Х^2 ^test with Yates correction, or Fisher's exact test, was used. The SPSS for Windows program (SPSS, Inc., Chicago, IL, version 17.0) was used for this analysis. All P-values were two-sided. Unless otherwise stated, data are expressed as mean ± s.e.m.

## Competing interests

The authors declare that they have no competing interests.

## Authors' contributions

MMC participated in the design of the experiments and carried out animal treatments, sample collection and processing (MMP activity assays, quantitative PCRs, Western blot analyses, immunofluorescence experiments and cell culture approaches) and statistical analyses. CA and EP-G participated in animal treatments, sample collection, quantitative PCRs and Western blot analyses. EM participated in the design of the study and carried out immunofluorescence experiments. CC participated in animal treatments and immunofluorescence experiments. JMF carried out the histopathological analyses of animal tumors and metastases. GM-B performed and analyzed the tissue microarrays of human samples. IG-R analyzed metastases in MMTV-neu mice by magnetic resonance imaging. JP carried out the histopathological characterization of human breast samples. SM participated in the design of the study. MG contributed to the conception and design of the study and critically revised the manuscript. CS conceived the study, elaborated its design, coordinated the research and wrote the manuscript. All authors read and approved the final manuscript.

## Supplementary Material

Additional file 1 **Figures S1-S5**. Supplemental Figure 1: MMTV-neu mice spontaneously develop breast tumors and lung metastases. Supplemental Figure 2: Cannabinoids inhibit breast tumor growth *in vivo *Supplemental Figure 3: MMTV-neu-derived tumors express cannabinoid receptors Supplemental Figure 4: Cannabinoids modulate the expression of MMP2 and MMP9 Supplemental Figure 5: Human ErbB2-positive breast cancer cell lines are sensitive to cannabinoids Click here for file

## References

[B1] Baselga J Swain SM Novel anticancer targets: revisiting ERBB2 and discovering ERBB3 Nat Rev Cancer 2009 9 463 475 10.1038/nrc2656 19536107

[B2] Ursini-Siegel J Schade B Cardiff RD Muller WJ Insights from transgenic mouse models of ERBB2-induced breast cancer Nat Rev Cancer 2007 7 389 397 10.1038/nrc2127 17446858

[B3] Di Cosimo S Baselga J Targeted therapies in breast cancer: where are we now? Eur J Cancer 2008 44 2781 2790 10.1016/j.ejca.2008.09.026 19013786

[B4] Hynes NE Lane HA ERBB receptors and cancer: the complexity of targeted inhibitors Nat Rev Cancer 2005 5 341 354 10.1038/nrc1609 15864276

[B5] Di Marzo V Targeting the endocannabinoid system: to enhance or reduce? Nat Rev Drug Discov 2008 7 438 455 10.1038/nrd2553 18446159

[B6] Pertwee RG Emerging strategies for exploiting cannabinoid receptor agonists as medicines Br J Pharmacol 2009 156 397 411 10.1111/j.1476-5381.2008.00048.x 19226257 PMC2697681

[B7] Guzman M Cannabinoids: potential anticancer agents Nat Rev Cancer 2003 3 745 755 10.1038/nrc1188 14570037

[B8] Sarfaraz S Adhami VM Syed DN Afaq F Mukhtar H Cannabinoids for cancer treatment: progress and promise Cancer Res 2008 68 339 342 10.1158/0008-5472.CAN-07-2785 18199524

[B9] Guy CT Webster MA Schaller M Parsons TJ Cardiff RD Muller WJ Expression of the neu protooncogene in the mammary epithelium of transgenic mice induces metastatic disease Proc Natl Acad Sci USA 1992 89 10578 10582 10.1073/pnas.89.22.10578 1359541 PMC50384

[B10] Chambers AF Groom AC MacDonald IC Dissemination and growth of cancer cells in metastatic sites Nat Rev Cancer 2002 2 563 572 10.1038/nrc865 12154349

[B11] Moasser MM The oncogene HER2: its signaling and transforming functions and its role in human cancer pathogenesis Oncogene 2007 26 6469 6487 10.1038/sj.onc.1210477 17471238 PMC3021475

[B12] Manning BD Cantley LC AKT/PKB signaling: navigating downstream Cell 2007 129 1261 1274 10.1016/j.cell.2007.06.009 17604717 PMC2756685

[B13] Nanni P Pupa SM Nicoletti G De Giovanni C Landuzzi L Rossi I Astolfi A Ricci C De Vecchi R Invernizzi AM p185(neu) protein is required for tumor and anchorage-independent growth, not for cell proliferation of transgenic mammary carcinoma Int J Cancer 2000 87 186 194 10.1002/1097-0215(20000715)87:2<186::AID-IJC5>3.0.CO;2-1 10861472

[B14] Dean-Colomb W Esteva FJ Her2-positive breast cancer: herceptin and beyond Eur J Cancer 2008 44 2806 2812 10.1016/j.ejca.2008.09.013 19022660

[B15] Hutchinson JN Jin J Cardiff RD Woodgett JR Muller WJ Activation of Akt-1 (PKB-alpha) can accelerate ErbB-2-mediated mammary tumorigenesis but suppresses tumor invasion Cancer Res 2004 64 3171 3178 10.1158/0008-5472.CAN-03-3465 15126356

[B16] Caffarel MM Moreno-Bueno G Cerutti C Palacios J Guzman M Mechta-Grigoriou F Sanchez C JunD is involved in the antiproliferative effect of Delta(9)-tetrahydrocannabinol on human breast cancer cells Oncogene 2008 27 5033 5044 10.1038/onc.2008.145 18454173

[B17] Caffarel MM Sarrio D Palacios J Guzman M Sanchez C Delta9-tetrahydrocannabinol inhibits cell cycle progression in human breast cancer cells through Cdc2 regulation Cancer Res 2006 66 6615 6621 10.1158/0008-5472.CAN-05-4566 16818634

[B18] De Petrocellis L Melck D Palmisano A Bisogno T Laezza C Bifulco M Di Marzo V The endogenous cannabinoid anandamide inhibits human breast cancer cell proliferation Proc Natl Acad Sci USA 1998 95 8375 8380 10.1073/pnas.95.14.8375 9653194 PMC20983

[B19] Laezza C Pisanti S Crescenzi E Bifulco M Anandamide inhibits Cdk2 and activates Chk1 leading to cell cycle arrest in human breast cancer cells FEBS Lett 2006 580 6076 6082 10.1016/j.febslet.2006.09.074 17055492

[B20] Ligresti A Moriello AS Starowicz K Matias I Pisanti S De Petrocellis L Laezza C Portella G Bifulco M Di Marzo V Antitumor activity of plant cannabinoids with emphasis on the effect of cannabidiol on human breast carcinoma J Pharmacol Exp Ther 2006 318 1375 1387 10.1124/jpet.106.105247 16728591

[B21] Qamri Z Preet A Nasser MW Bass CE Leone G Barsky SH Ganju RK Synthetic cannabinoid receptor agonists inhibit tumor growth and metastasis of breast cancer Mol Cancer Ther 2009 8 3117 3129 10.1158/1535-7163.MCT-09-0448 19887554 PMC4128286

[B22] Melck D De Petrocellis L Orlando P Bisogno T Laezza C Bifulco M Di Marzo V Suppression of nerve growth factor Trk receptors and prolactin receptors by endocannabinoids leads to inhibition of human breast and prostate cancer cell proliferation Endocrinology 2000 141 118 126 10.1210/en.141.1.118 10614630

[B23] Melck D Rueda D Galve-Roperh I De Petrocellis L Guzman M Di Marzo V Involvement of the cAMP/protein kinase A pathway and of mitogen-activated protein kinase in the anti-proliferative effects of anandamide in human breast cancer cells FEBS Lett 1999 463 235 240 10.1016/S0014-5793(99)01639-7 10606728

[B24] Frese KK Tuveson DA Maximizing mouse cancer models Nat Rev Cancer 2007 7 645 658 10.1038/nrc2192 17687385

[B25] Wang D Wang H Ning W Backlund MG Dey SK DuBois RN Loss of cannabinoid receptor 1 accelerates intestinal tumor growth Cancer Res 2008 68 6468 6476 10.1158/0008-5472.CAN-08-0896 18676872 PMC2561258

[B26] Izzo AA Aviello G Petrosino S Orlando P Marsicano G Lutz B Borrelli F Capasso R Nigam S Capasso F Di Marzo V Increased endocannabinoid levels reduce the development of precancerous lesions in the mouse colon J Mol Med 2008 86 89 98 10.1007/s00109-007-0248-4 17823781 PMC2755791

[B27] Lopez-Otin C Matrisian LM Emerging roles of proteases in tumour suppression Nat Rev Cancer 2007 7 800 808 10.1038/nrc2228 17851543

[B28] Mira E Lacalle RA Buesa JM de Buitrago GG Jimenez-Baranda S Gomez-Mouton C Martinez AC Manes S Secreted MMP9 promotes angiogenesis more efficiently than constitutive active MMP9 bound to the tumor cell surface J Cell Sci 2004 117 1847 1857 10.1242/jcs.01035 15075244

[B29] Scorilas A Karameris A Arnogiannaki N Ardavanis A Bassilopoulos P Trangas T Talieri M Overexpression of matrix-metalloproteinase-9 in human breast cancer: a potential favourable indicator in node-negative patients Br J Cancer 2001 84 1488 1496 10.1054/bjoc.2001.1810 11384099 PMC2363667

[B30] Pisanti S Borselli C Oliviero O Laezza C Gazzerro P Bifulco M Antiangiogenic activity of the endocannabinoid anandamide: correlation to its tumor-suppressor efficacy J Cell Physiol 2007 211 495 503 10.1002/jcp.20954 17192847

[B31] Blazquez C Salazar M Carracedo A Lorente M Egia A Gonzalez-Feria L Haro A Velasco G Guzman M Cannabinoids inhibit glioma cell invasion by down-regulating matrix metalloproteinase-2 expression Cancer Res 2008 68 1945 1952 10.1158/0008-5472.CAN-07-5176 18339876

[B32] Decock J Paridaens R Cufer T Proteases and metastasis: clinical relevance nowadays? Curr Opin Oncol 2005 17 545 550 10.1097/01.cco.0000180435.39614.63 16224231

[B33] Jezierska A Motyl T Matrix metalloproteinase-2 involvement in breast cancer progression: a mini-review Med Sci Monit 2009 15 RA32 40 19182722

[B34] Minn AJ Gupta GP Siegel PM Bos PD Shu W Giri DD Viale A Olshen AB Gerald WL Massague J Genes that mediate breast cancer metastasis to lung Nature 2005 436 518 524 10.1038/nature03799 16049480 PMC1283098

[B35] Guzman M Duarte MJ Blazquez C Ravina J Rosa MC Galve-Roperh I Sanchez C Velasco G Gonzalez-Feria L A pilot clinical study of Delta9-tetrahydrocannabinol in patients with recurrent glioblastoma multiforme Br J Cancer 2006 95 197 203 10.1038/sj.bjc.6603236 16804518 PMC2360617

[B36] Natrajan R Lambros MB Rodriguez-Pinilla SM Moreno-Bueno G Tan DS Marchio C Vatcheva R Rayter S Mahler-Araujo B Fulford LG Tiling path genomic profiling of grade 3 invasive ductal breast cancers Clin Cancer Res 2009 15 2711 2722 10.1158/1078-0432.CCR-08-1878 19318498

