# Supplementary material for: Cannabinoids reduce ErbB2-driven breast cancer progression through Akt inhibition
Source: Mol Cancer. 2010 Jul 22;9:196. doi: 10.1186/1476-4598-9-196 (PMC2917429; doi:10.1186/1476-4598-9-196)
Supplement: Additional file 1 — Figures S1-S5. Supplemental Figure 1: MMTV-neu mice spontaneously develop breast tumors and lung metastases. Supplemental Figure 2: Cannabinoids inhibit breast tumor growth in vivo Supplemental Figure 3: MMTV-neu-derived tumors express cannabinoid receptors Supplemental Figure 4: Cannabinoids modulate the expression of MMP2 and MMP9 Supplemental Figure 5: Human ErbB2-positive breast cancer cell lines are sensitive to cannabinoids [file 1476-4598-9-196-S1.PDF]

MM Caffarel *et al.*
Supplemental Figure 1

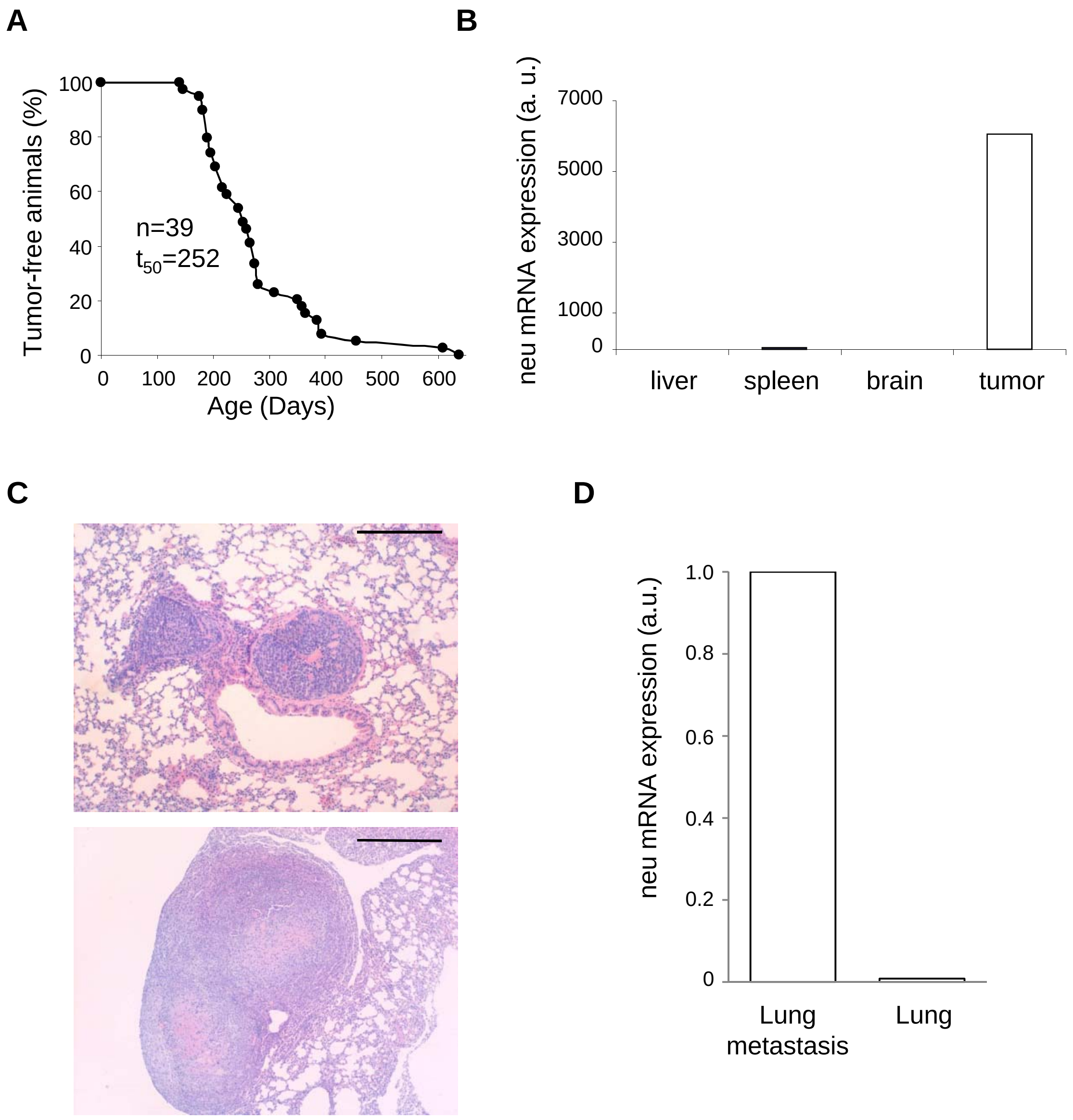

Figure S1. MMTV-neu mice spontaneously develop breast tumors and lung metastases. (A) Kinetics of breast tumor occurrence in MMTV-neu females. (B) Rat ErbB2 (neu transgene) mRNA expression in different MMTV-neu mouse organs, as determined by real-time quantitative PCR. (C) Representative images of a small (upper panel) and a big (lower panel) lung metastasis. Scale bars: 200 µm (upper panel) and 100 µm (lower panel) . (D) Rat ErbB2 (neu transgene) mRNA expression in a representative lung metastasis and the surrounding healthy lung tissue, as determined by real-time quatitative-PCR. Data are expressed in arbitrary units.

MM Caffarel *et al.*
Supplemental Figure 2

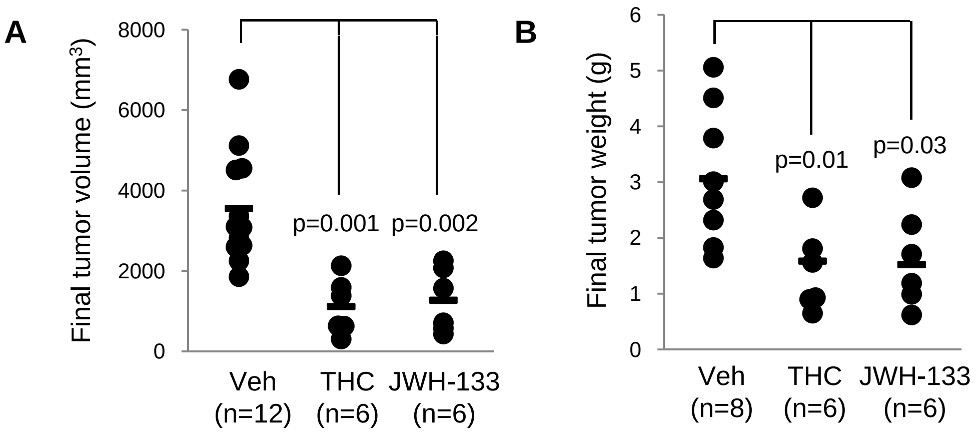

Figure S2. Cannabinoids inhibit breast tumor growth *in vivo*. Final volume (A) and final weight (B) of the first tumor appeared in each animal.

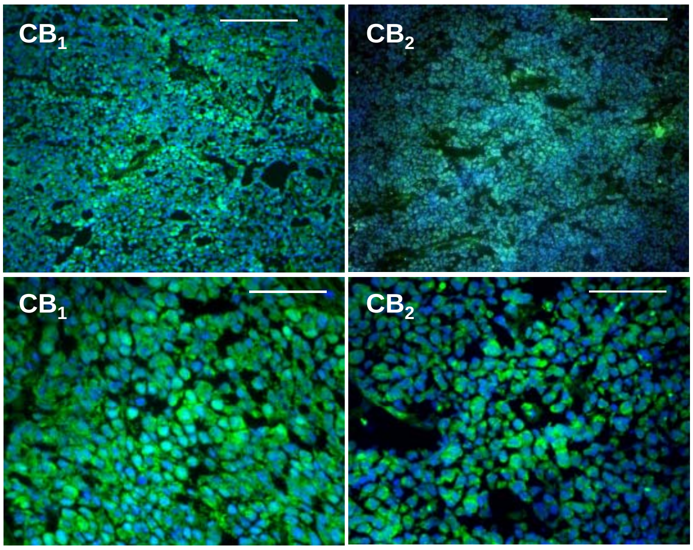

Figure S3. MMTV-neu-derived tumors express cannabinoid receptors. CB_1 receptor and CB_2 receptor expression (green) in tumor sections. Cell nuclei are in blue. Scale bars: upper panels, 120 µm; lower panels, 40 µm.

MM Caffarel *et al.*
Supplemental Figure 4

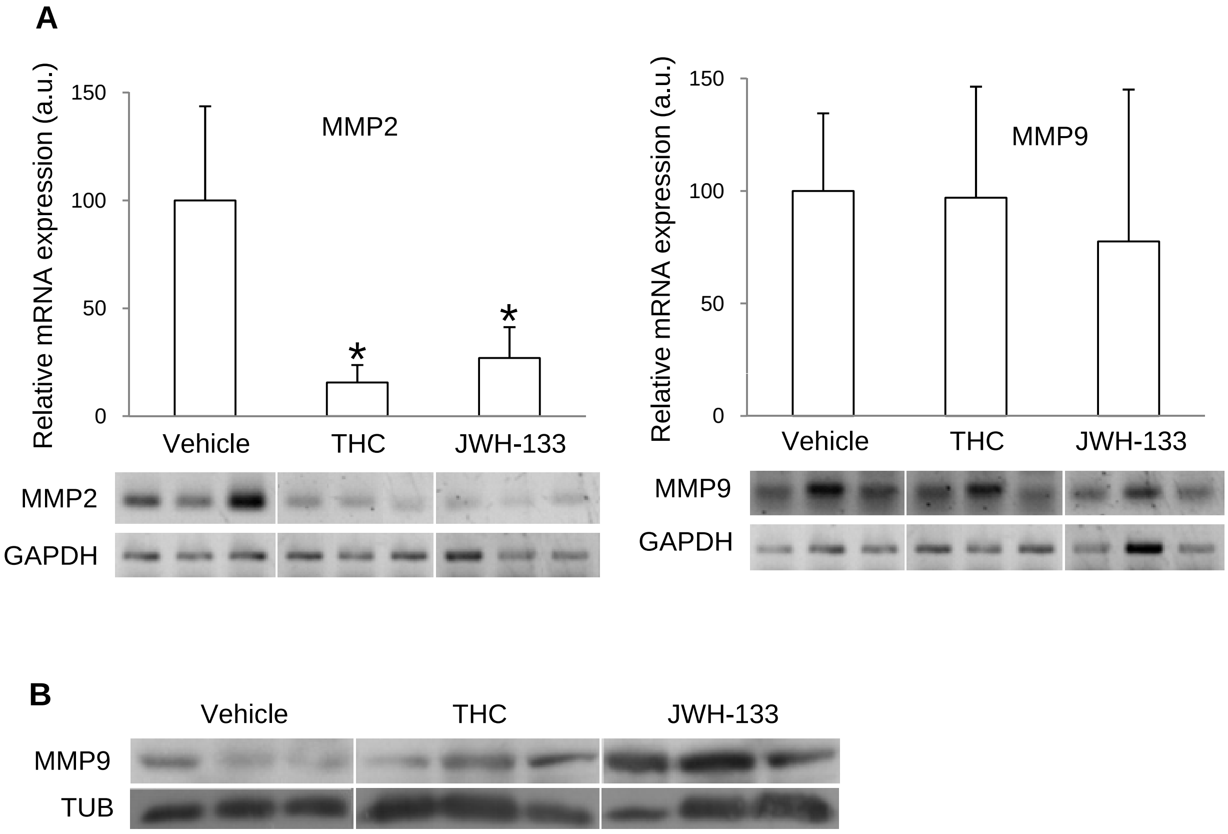

Figure S4. Cannabinoids modulate the expression of MMP2 and MMP9. (A) MMP2 (left panel) and MMP9 (right panel) mRNA expression in vehicle-treated, THC-treated and JWH-133-treated MMTV-neu mice, as determined by real-time quantitative PCR (graphs) and reverse-transcriptase PCR (pictures; 3 representative tumors of each group are shown). Data are expressed in arbitrary units. (B) MMP9 protein expression, as determined by Western blot, in the same tumors. *, $p<0.05$ *vs* vehicle-treated tumors.

MM Caffarel *et al.*
Supplemental Figure 5

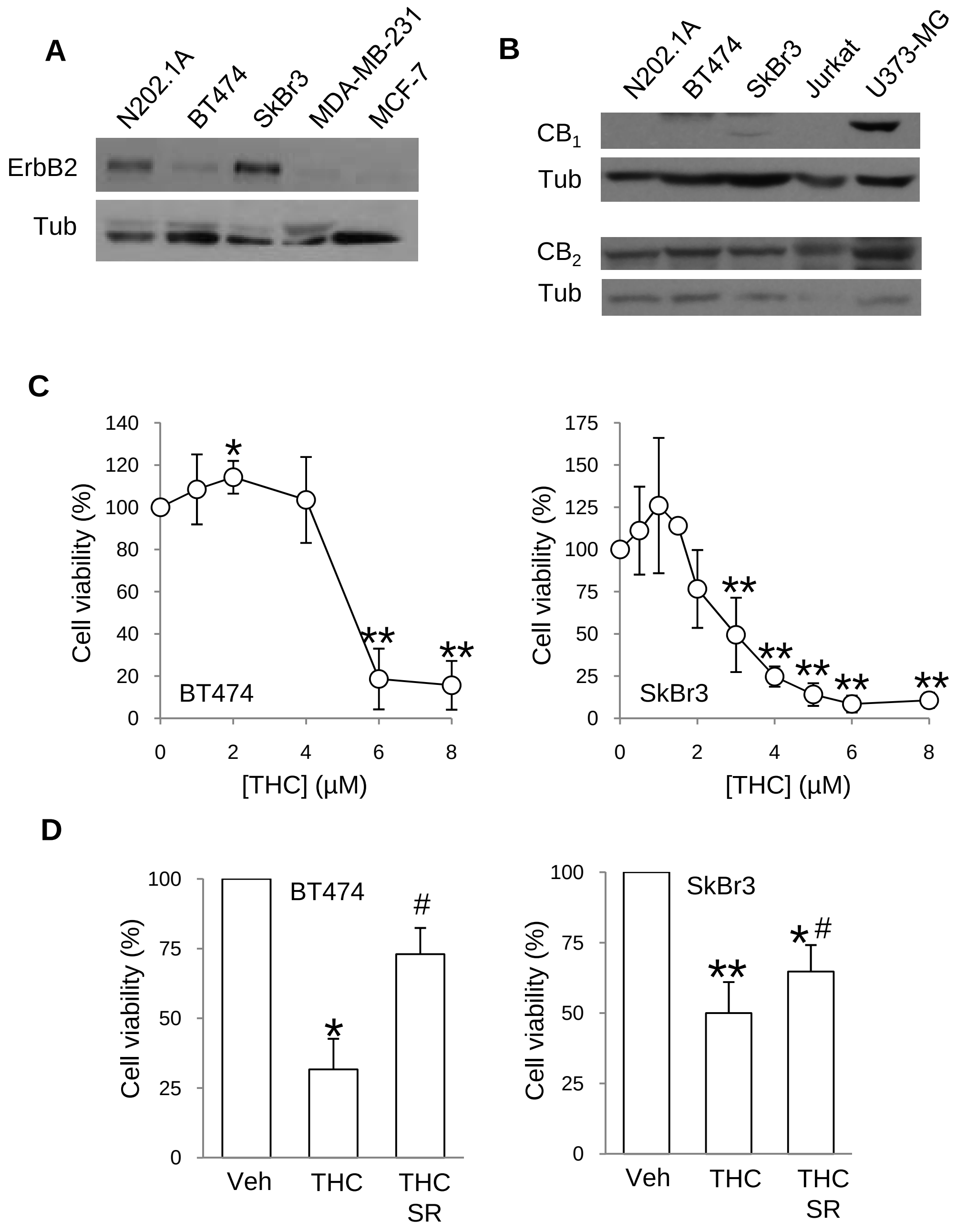

Figure S4. Human ErbB2-positive breast cancer cell lines are sensitive to cannabinoids. (A) ErbB2 and (B) CB_1 and CB_2 receptor expression, as determined by Western blot, in different breast cancer cell lines from mouse (N202.1A) and human (BT474, SkBr3, MDA-MB-231 and MCF-7) origin. MDA-MB-231 and MCF-7 cells were used as ErbB2-negative controls. U373-MG and Jurkat cells were used as positive controls for CB_1 and CB_2 receptor expression, respectively. (C and D) Viability of BT474 and SkBr3 cells in response to (C) increasing concentrations of THC or (D) 6 µM (BT474) or 3 µM THC (SkBr3) with or without 2 µM SR144528 (SR) for 72 h. Data are expressed as % of vehicle-treated cells, set at 100%. *, p<0.05; **, p<0.01 *vs* vehicle-treated cells; #, p<0.05 *vs* THC-treated cells.
